# Robust image hashing using ring partition-PGNMF and local features

**DOI:** 10.1186/s40064-016-3639-6

**Published:** 2016-11-21

**Authors:** Ram Kumar Karsh, R. H. Laskar, Bhanu Bhai Richhariya

**Affiliations:** 1 Department of Electronics and Communication Engineering, NIT Silchar, Silchar, Assam 788010 India; 2 Department of Electronics and Communication Engineering, NIT Mizoram, Aizawl, Mizoram 796012 India

**Keywords:** Image hashing, Multimedia security, PGNMF, Ring partition, Saliency detection

## Abstract

**Background:**

Image authentication is one of the challenging research areas in the multimedia technology due to the availability of image editing tools. Image hash may be used for image authentication which should be invariant to perceptually similar image and sensitive to content changes. The challenging issue in image hashing is to design a system which simultaneously provides rotation robustness, desirable discrimination, sensitivity and localization of forged area with minimum hash length.

**Methods:**

In this paper, a perceptually robust image hashing technique based on global and local features has been proposed. The Global feature was extracted using ring partition and projected gradient nonnegative matrix factorization (PGNMF). The ring partitioning technique converts a square image into a secondary image that makes the system rotation invariant. The PGNMF which is usually faster than the other NMFs has been used to reduce the dimension of the secondary image to generate the shorter hash sequence. The local features extracted from the salient regions of the image help to localize the forged region in the maliciously manipulated images. The image hashing techniques that use only global features are limited in discrimination.

**Results:**

The experimental results reveal that the proposed image hashing method based on global and local features provides better discrimination capability. The proposed hashing method is tested on large image sets collected from the different standard database. It is observed from the experimental results that the proposed system is robust to content-preserving operations and is capable of localizing the counterfeit area.

**Conclusions:**

The combination of global and local features is robust against the content-preserving operations, which has a desirable discriminative capability. The proposed system may be used in image authentication, forensic evidence, and image retrieval, etc.

## Background

The extensive use of the Internet and multimedia services has made the image and video processing to be a part of our day-to-day activities. On the other hand, the academia and industries are facing several challenges due to the malicious manipulations of the images using image editing tools. Recognizing similar versions of the image from huge multimedia records is still a challenging issue (Wu et al. 2007). Attempt to overcome these challenge has led to an emerging multimedia technology known as image hashing. It is a technique that extracts a short sequence from the image to represent its contents, which is used for various image processing applications (Ahmed et al. 2010; Hassan et al. 2012; Lu et al. 2004; Lu and Wu 2010; Lv and Wang 2009; Qin et al. 2012; Slaney and Casey 2008; Tang et al. 2008).

Hash function serves as an effective tool for message authentication in cryptographic applications. The hash function [such as a message digest-5 (MD-5) and a secure hash algorithm-1 (SHA-1)] defines a mapping from an arbitrary-length message to a short digest (i.e. hash string) (Stamp 2006). The prerequisite condition to accomplish the cryptographic authentication using a hashing is the avalanche effect, in which a single bit modification on the message may lead to the significant changes in the hash string. However, the over-sensitivity of the cryptographic hash function limits its applications in the multimedia domain. In the multimedia applications, the hash function should be robust against the content-preserving operations, such as the geometric transformation, format conversion, and an image compression, etc. In other words, the hash of original image and its perceptually similar version should be approximately same. Hash should be considerably different only when the visual content is distorted by the malevolent process such as object deletion/insertion operations etc. One more important property is the discriminative capability, i.e. images with dissimilar content should provide different hashes.

Various state-of-the-art image hashing methods have been proposed in the literature. Monga et al. (2006) proposed a two-stage method which consists of feature extraction and dimension reduction using clustering technique to design the final hash. It has become a commonly adopted approach for further developing the image hashing techniques. The state-of-the-art methods are either based on global (Lei et al. 2011; Swaminathan et al. 2006; Tang et al. 2008; Xiang et al. 2007) or local features (Ahmed et al. 2010; Fouad and Jianmin 2010; Khelifi and Jiang 2010; Lv and Wang 2012; Monga and Mihcak 2007; Tang et al. 2011). The image hashing techniques based on only the global feature generally provides shorter hash length, but fails to identify the local changes in the image. On the other hand, the image hashing techniques based on only the local features are generally sensitive to local changes in the image, but provides longer hash length. Xiang et al. (2007) developed an image hashing technique using image histogram that is invariant to geometric transformations. This method generally misclassifies the different images having similar histograms. Tang et al. (2008) proposed an image hashing algorithm using nonnegative matrix factorization (NMF). In this method, the secondary image is obtained by pseudo-randomly re-arranging the pixels of the normalized image. The secondary image is then passed through the NMF, the computed coefficients are then quantized and scrambled to generate the final hash. Swaminathan et al. (2006) developed a Fourier-Mellin transform based method which is rotation invariant and provides better security. Lei et al. (2011) proposed an image hashing technique based on discrete Fourier transform (DFT) and radon transform (RT). DFT is carried out on the invariant moments generated by the Radon transformation of the original image. The computed DFT coefficients are then quantized to form the final hash. Khelifi and Jiang (2010) calculated image hash on the basis of the virtual watermark detection, which is robust for the geometric deformations and content preserving operations. This method can detect only the changes in large areas. An NMF–NMF based technique has also been proposed to generate image hash (Monga and Mihcak 2007). First NMF provides a secondary image from pseudo-randomly selected sub-images and the second NMF is used to generate the low-rank matrix. Then the matrix entries are concatenated to form the NMF–NMF vector which is considered to be the final hash. This method fails to identify the counterfeit areas. An analysis of NMF–NMF method suggests that out of the three keys, the first one used for pseudo-randomly selecting sub-images is only important (Fouad and Jianmin 2010).

Tang et al. (2011) proposed a lexicographical-framework for image hashing that uses discrete cosine transform (DCT) and NMF. This method is robust to some content preserving operations, but fragile to large degree rotation. Ahmed et al. (2010) has developed an image hashing using discrete wavelet transform (DWT) and SHA-I. It has been used for tamper detection, but limited in brightness/contrast changes and rotation. An image hashing technique based on scale invariant feature transform (SIFT) and Harris detector has also been proposed Lv and Wang (2012). In this method, key points are extracted from the scale space image by using SIFT and from which the stable key points are retained by Harris detector. The shape-texture features around the stable key points are used to generate the final hash. It is robust to the geometric deformations and can also find the counterfeit area. This method does not possess rotation invariant property.

Lu et al. (2010) developed a concept of forensic hash for information assurance. It is based on RT and the scale space theory. For image authentication, it uses the side information. This method can provide the history of image manipulations and parameters for geometric deformation. It can also identify the counterfeit area. An image hashing method with the combination of global and local features has been proposed in the literature (Zhao et al. 2013). These features are based on Zernike moments and shape-texture respectively. It is robust to the most content preserving operations and can also locate the forged areas. But it is not robust to the large degree of rotation.

 Tang et al. (2016a, b) proposed an image hashing based on ring partition and invariant vector distance by incorporating invariant vector distance in place of NMF. This method is robust against perceptual digital image manipulation, but it cannot localize the forged region. The entropy based method (Tang et al. 2013) creates a rotation invariant secondary image based on the ring partition and extracts entropy from each ring to generate the image hash. This method is robust to digital image manipulations and possesses good discrimination capability for the natural image, but limited discrimination capability for textural images.

Sun and Zeng (2014) designed an image hashing based on combining the Fourier-Mellin transform (FMT) and the compressive sensing (CS). This method is resilient to scaling, and transition attacks, but sensitive to large angle rotation. The global features extracted from partitioned image blocks and local feature extracted from key points of the image have been concatenated to form an image hash (Wang et al. 2015). It is robust to perceptual image manipulation, can locate the forged area but sensitive to large angle rotation. An image hashing method based on the quaternion discrete Fourier transform followed by log-polar transform has been developed (Ouyang et al. 2015). In this method, the three colors have been used to generate hash without increasing the hash length. This method is incapable of locating the counterfeit areas in the forged image. Yan et al. (2016) proposed an image hashing method using adaptive local features. It is robust against the content-preserving operations, but unable to detect color forgery. Qin et al. (2016) has designed a hashing method based on the block truncation coding (BTC). The major drawback of this method is sensitive towards brightness changes. An image hashing technique based on ring partition and color vector angle has been proposed by Tang et al. (2016a, b). This method is robust to digital image manipulation, but around 20% of the corner information has not been utilized to design the image hash.

Image rotation is a normal content preserving operation that is considered to be a major challenging issue in the case of image hashing. Most of the conventional image hashing methods categorize the rotated images as different images (Fridrich and Goljan 2000; Qin et al. 2012; Lin and Chang 2001; Tang et al. 2011; Venkatesan et al. 2000; Wu et al. 2009). There are some existing methods that are resilient to the rotation but provides the poor discriminative capability (Kozat et al. 2004; Lefebvre et al. 2002; Lei et al. 2011; Monga and Mihcak 2007; Roover et al. 2005; Swaminathan et al. 2006). Even though the ring partition and NMF based method (Tang et al. 2014) having good discrimination capability, is invariant to arbitrary rotation, yet it cannot localize the counterfeit region of the forged image. Localization of the counterfeit regions in the forged image is very useful in the medical and forensic evidence (Mishra and Adhikary 2013). This method also suffers from the convergence problem of the matrix factorization, which is based on the multiplicative update method. It is an extremely challenging task to design an image hashing technique that simultaneously satisfies all the requirements, such as rotation robustness, good discriminative capability, and localization of the forged area.

In this paper, a robust image hashing technique has been proposed to meet the above requirements simultaneously. In the proposed method, the secondary image obtained through ring portioning makes the system rotation invariant. The use of PGNMF instead of NMF helps the system to converge faster. The attribute of the PGNMF (i.e. learning parts-based representation) and use of the local features along with the global features helps to enhance the discrimination capability of the proposed algorithm. The combination of local features with the global features also makes the system capable of identifying the forged regions in the manipulated images.

Experiments were carried out on large image sets that were taken from standard image databases (USC-SIPI Image Database 2007; Ground Truth Database 2008) and downloaded from the internet. The experimental results show that the proposed algorithm provides better tradeoff amongst the rotation robustness, discrimination capability and localizing the counterfeit region.

## Methods

**Fig. 1:**
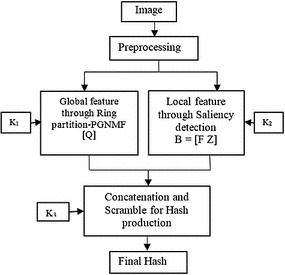
Block diagram of the proposed image hashing

The proposed image hashing involves four different steps as shown in Fig. 1.

The input image is transformed to a normalized image size (256 × 256) with pre-processing. The final hash is formed by concatenating the two intermediate hash. First intermediate hash, is generated from the global features derived from the normalized image using ring partitioning and PGNMF. Second intermediate hash is generated from the shape-texture feature derived from the salient regions of the image (Hou and Zhang 2007). The final hash has been scrambled using the secret key K_3_. The PGNMF, ring partition, saliency detection scheme, local features and the proposed algorithm in details with a probable application have been discussed in the subsequent subsections.

### PGNMF

Non-negative matrix factorization is an algorithm where a matrix is factorized into two matrices of lower dimension, with the property that all matrices have only positive elements (Paatero and Tapper 1994). Let ${\mathbf{L}}$ be a matrix of size *m* × *n*, where *L*_*ij*_ ≥ 0, then it can be approximately represented by the two non-negative matrices ${\mathbf{W}} \in {\mathbf{R}}^{m \times p}$ (i.e. base matrix) and ${\mathbf{H}} \in {\mathbf{R}}^{p \times n}$ (i.e. coefficient matrix) by using NMF such as:

1 $${\mathbf{L}} \approx {\mathbf{WH}}$$

where *p* is the rank of NMF that should satisfy the condition $p < { \hbox{min} }\left( {m,n} \right)$.

If each column of the matrix ${\mathbf{L}}$ represents the objects of an image, then NMF approximate it by linear combination of *p* basis columns in ${\mathbf{W}}$. There are various applications of NMF such as document clustering (Xu et al. 2003), finding basis vectors of the images (Lee and Seung 2001), and molecular pattern discovery (Brunet et al. 2003). The theoretical issues related to NMF scheme have been discussed in Donoho and Stodden (2004). The state-of-the-art NMF schemes have the convergence problem. To overcome this problem, Lin (2007) proposed a method, named PGNMF which has the faster optimization property. The illustration of PGNMF scheme is shown in Table 1.

**Table 1:**
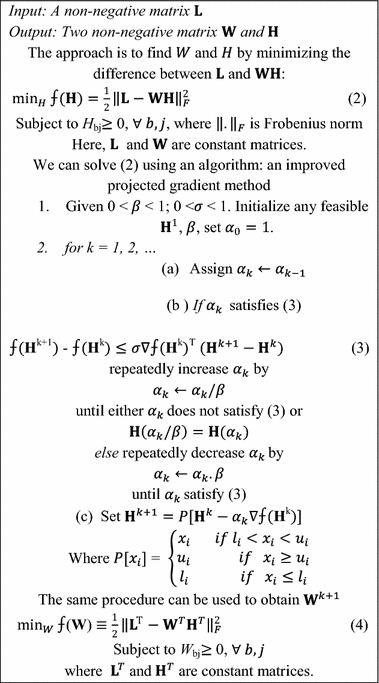
The PGNMF algorithm

### Square image to secondary image (circular) conversion

**Fig. 2:**
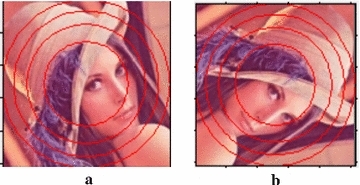
Depicts the similar image information in rings of original image and its rotated version. **a** Original image. **b** 90° rotated image

**Fig. 3:**
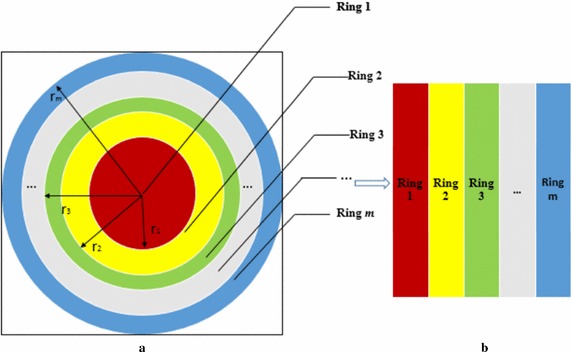
Ring partition of a square image to construct secondary image. **a** ​​Formation of annular rings (*Red-Ring* *1*, *Yellow-Ring* *2*, *Green-Ring* *3*,...., *Blue-Ring* *m*). **b** Corresponding matrix representation

Generally, image center remains fix after rotation manipulation. It has been observed that pixel values in different rings of the original image and its rotated version remain same as shown in Fig. 2a, b respectively. Hence, the square image can be converted into rings that may be subsequently used to construct the secondary image as shown in Fig. 3. Use of this concept makes the proposed system rotation invariant (Tang et al. 2014). The secondary image is constructed as follows:

Let the size of a square image be *n* × *n*, the number of rings is *m*, and ${\mathbf{P}}_{i}$ be the set of pixel values in the *i*_*th*_ ring $\left( {i = 1, 2, \ldots ,m} \right)$. The inscribed circle area from a square image has been used for hash generation. The inscribed area has been divided into annular rings of equal area as shown in Fig. 3a. Each ring provides the column of the secondary image as shown in Fig. 3b. The radii of circles can be determined as follows: For an image of size *n* × *n*, the outermost radius is $r_{m} = \left[ {\frac{n}{2}} \right]$. To find the other radii, firstly the area *S* enclosed by outermost radius is calculated and then the other ring area U_A_, i.e. average area is found as follows (Tang et al. 2014):

5 $$S = \pi r_{m}^{2}$$

6 $$U_{A} = \left[ {S/m} \right]$$

So, $r_{1}$ can be computed by

7 $$r_{1} = \sqrt {\frac{{U_{A} }}{\pi }}$$

Thus, other radii $r_{k} \left( {k = 1, 2, \ldots ,m - 1} \right)$, can be found using the following equation:

8 $$r_{k} = \sqrt {\frac{{U_{A} + \pi r_{k - 1}^{2} }}{\pi }}$$

The process of arranging the pixels from the rings (shown in Fig. 3a) into columns (shown in Fig. 3b) is as follows: Let the coordinates of the image center be (*x*_*c*_, *y*_*c*_). If *n* is an even number, then, $x_{c} = \frac{n}{2} + 0.5$ and $y_{c} = \frac{n}{2} + 0.5$, otherwise, $x_{c} = \frac{{\left( {n + 1} \right)}}{2}$ and $y_{c} = \frac{{\left( {n + 1} \right)}}{2}$.

A circular region (${\mathbf{C}}_{1}$) (i.e. Ring 1) of radius *r*_1_ around the center pixel has been selected as shown in (9)

9 $${\mathbf{C}}_{1} : \left( {x - x_{c} } \right)^{2} + \left( {y - y_{c} } \right)^{2} = r_{1}^{2}$$

where *y*th row and the *x*th column of the normalized image is given by $1 \le x \le n, \;1 \le y \le n$.

**Fig. 4:**
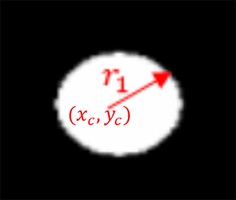
First Mask $({\mathbf{T}}_{1} )$ for ${\text{r}}_{1}$

The process of creating a mask $({\mathbf{T}}_{1} )\varvec{ }$ for the first radius *r*_1_ is given below:

First mask $({\mathbf{T}}_{1} )$ is constructed having pixel value one (*true*) inside the ring of radius *r*_1_ and pixel value zero (*false*) outside the circle as shown in Fig. 4.

Let the pixel values of this region (i.e. Ring 1) be placed in the first column of matrix **L** by multiplying the input image $\left( {\mathbf{I}} \right)$ and first mask $({\mathbf{T}}_{1} )$:

10 $${\mathbf{L}}_{1} = {\mathbf{I}} \times {\mathbf{T}}_{1} ;\quad {\text{inside r}}_{1}$$

Similarly, other circles ${\mathbf{C}}_{k} \left( {k = 2,3, \ldots ,\,m} \right)$ can be constructed for *r*_*k*_(*k* = 2, 3, …, *m*).

11 $${\mathbf{C}}_{k} :\left( {x - x_{c} } \right)^{2} + \left( {y - y_{c} } \right)^{2} = r_{k}^{2}$$

**Fig. 5:**
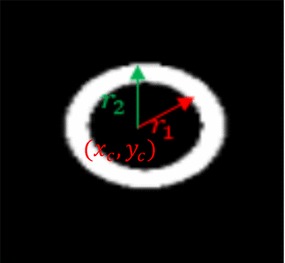
Second mask $({\mathbf{T}}_{2} )$ for $({\mathbf{r}}_{2} - {\mathbf{r}}_{1} )$

The second mask $({\mathbf{T}}_{2} )$ is constructed with pixel value one (*true*) for the pixels inside the annular ring of *r*_1_ ≤ *r* ≤ *r*_2_ and pixel value zero (*false*) outside the annular ring as shown in Fig. 5.

**Fig. 6:**
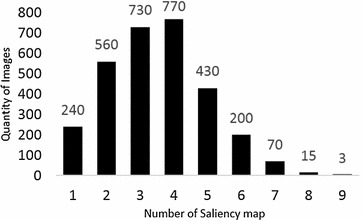
Representation of saliency map

The pixel values of this region is placed (i.e. annular Ring 2) into the second column of matrix **L** by multiplying the input image $\left( {\mathbf{I}} \right)$ and second mask $({\mathbf{T}}_{2} )$:

12 $${\mathbf{L}}_{2} = {\mathbf{I}} \times {\mathbf{T}}_{2} ;\quad {\text{for}}\,(\varvec{r}_{2} - \varvec{r}_{1} )$$

Similarly, pixel values of other regions (i.e. annular Rings) are putted in the other column of matrix L:

13 $${\mathbf{L}}_{k} = {\mathbf{I}} \times {\mathbf{T}}_{k} ;\quad {\text{for}}\,(\varvec{r}_{k} - \varvec{r}_{k - 1} )$$

Thus, the rotation invariant matrix is obtained as follows:

14 $${\mathbf{L}} = \left[ {{\mathbf{L}}_{1} ,\;{\mathbf{L}}_{2} ,\;{\mathbf{L}}_{3} , \ldots ,\;{\mathbf{L}}_{m} } \right]$$

To compress a high dimensional vector, the matrix **L** is processed through PGNMF to obtain a compact form of first intermediate hash (**Q**).

### Saliency map recognition

The salient region is defined as the visual attention of any image. For every image, there are two parts, an innovation and a prior knowledge (Hou and Zhang 2007). It is also called the novel and redundant parts. Redundant parts should be removed to get the novel parts of an image.

The log-spectrum, $V\left( f \right)$ characterize the common information of an image. The redundant information is present in *V*(*f*) due to the fact that the log-spectra of different images are similar. The convolution of *V*(*f*) and an $l \times l$ low-pass kernel is used to find the redundant information $R\left( f \right)$, as given by (15)

15 $$R\left( f \right) = h_{l} \times V\left( f \right)$$

The novel portion (*N*(*f*)) of an image can be obtained by spectral residual method that can be derived by subtracting *R*(*f*) from *V*(*f*) and the salient map can be obtained by inverse Fourier transform of (*f*):

16 $$S_{m} \left( y \right) = {\text{F}}^{ - 1} \left[ {N\left( f \right)} \right] = {\text{F}}^{ - 1} \left[ {V\left( f \right) - R\left( f \right)} \right]$$

The salient region of an image can be obtained by taking the threshold three times of the mean of the $S_{m} \left( y \right)$ (Zhao et al. 2013).

### Local features

For human visual perception, the important local feature is the texture of the image. Six texture features have been proposed in Deselaers et al. (2004), Tamura et al. (1978). We have used three features, namely, coarseness (*δ*_1_), contrast (*δ*_2_), and kurtosis (*δ*_3_). To calculate coarseness with respect to a particular pixel(*i*, *j*), the average of its neighborhood size (2^*k*^ × 2^*k*^) is considered as follows:

17 $$A_{k} \left( {i,j} \right) = \frac{1}{{2^{2k} }}\mathop \sum \limits_{{x = i - 2^{k} }}^{{i + 2^{k} - 1}} \mathop \sum \limits_{{y = j - 2^{k} }}^{{j + 2^{k} - 1}} g\left( {x,y} \right),\quad k = 1,2, \ldots ,5$$

Here, $g\left( {x, y} \right)$ represents the gray-level pixel. At every pixel(*i*, *j*), differences of average values in vertical and horizontal direction is as follows:

18 $$E_{k,h} \left( {i,j} \right) = \left| {A_{k} \left( {i + 2^{k - 1} ,j} \right) - A_{k} \left( {i - 2^{k - 1} ,j} \right)} \right|$$

19 $$E_{k,v} \left( {i,j} \right) = \left| {A_{k} \left( {i,j + 2^{k - 1} } \right) - A_{k} \left( {i,j - 2^{k - 1} } \right)} \right|$$

The *S*_*best*_(*i*, *j*) represents the highest difference value evaluated at(*i*, *j*), and is given by (20)

20 $$S_{best} \left( {i,j} \right) = \mathop {\arg \hbox{max} }\limits_{k = 0, \ldots ,5;d = h,v} E_{k,d} \left( {i,j} \right)$$

The coarseness (*δ*_1_) for the image can be calculated as

21 $$\delta_{1} = \frac{1}{m \times n}\mathop \sum \limits_{i}^{m} \mathop \sum \limits_{j}^{n} S_{best} \left( {i,j} \right)$$

where, *m* and *n* represents the dimension of the image.

Image illumination changes are described as contrast. This can be evaluated using the variance (*σ* ^2^) and the fourth order moment (*μ*_4_) of the gray value for the given region.

22 $$\delta_{2} = \sigma^{2} \mu_{4}^{ - 1/4}$$

The kurtosis which is used to calculate polarization of image can be measured as

23 $$\delta_{3} = \frac{{\mu_{4} }}{{\sigma^{4} }}$$

### Brief description of the proposed image hashing

The detail of the proposed image hashing has been illustrated in Table 2.

**Table 2:** Proposed image hashing in details (From input image to generation of final hash)

	*Input: Image*	
1.	Pre-processing: The input image (**I**) is rescaled to a fixed size *n* × *n* by bilinear interpolation, and changed from the RGB to the YCbCr representation. The Y and $\left| {{\text{Cb}} - {\text{Cr}}} \right|$, known as luminance and chrominance constituents of the image which may be used to create the hash. However, we have selected *n* = 256 and Y channel only for this study	
2.	Global features extraction using ring partition-PGNMF	
	a.	Let us consider the Y component of the pre-processedimage;
	b.	Divide Y into *m* rings and produce a secondary image ${\mathbf{L}}$ by using Eqs. (5–14)
	c.	Apply PGNMF to **L**, and obtain the coefficient matrix ${\mathbf{H}}$
	d.	Concatenate the matrix coefficients to give the hash of length ${\mathbf{Q^{\prime}}} = nk$ by global feature
	e.	A secret key K_1_ randomly generates a row vector ${\mathbf{X}}_{1}$. The first intermediate hash$\left( {\mathbf{Q}} \right)$ is formulated as ${\mathbf{Q}} = \left[ {\left( {{\mathbf{Q^{\prime}}} + {\mathbf{X}}_{1} } \right) {\text{mod }}256} \right]$
3.	Extraction of local features	
	a.	Detect *C* numbers of largest salient regions from pre-processed image Y
	b.	The *C* numbers of position vectors of four dimensions are formed using the coordinates of the top left corner, and width/height of each rectangle around the salient regions. It is denoted by position vector ${\mathbf{f}}^{k} \left( {k = 1, 2, \ldots , C} \right)$
	c.	The texture features are computed for *C* number of salient regions. Fig. 6 shows that 96% of the images have less than 6 salient regions. We have used 2900 images to test the number of salient regions as shown in Fig. 6. Only a few of the images have more than 6 salient regions. There is a trade-off between the hash length and number of salient regions. When the value of *C* is large, fewer salient regions will be left out but will produce a longer image hash. We have taken *C* = 6, empirically as an optimal trade-off between hash length and the salient maps that will be left out. For every salient region, local texture features, that include coarseness (*δ*_1_), contrast (*δ*_2_) and kurtosis (*δ*_3_) are computed and rounded to form *C* element texture vectors ${\mathbf{z}}^{k} \left( {k = 1, 2, \ldots , C} \right)$ of three dimensions
	d.	The position vector $\left( {\mathbf{F}} \right)$ is concatenated with the texture vector $\left( {\mathbf{Z}} \right)$ to give local feature vector ${\mathbf{B^{\prime}}} = \left[ {{\mathbf{F}} {\mathbf{Z}}} \right] = \left[ {{\mathbf{f}}^{1} \ldots {\mathbf{f}}^{C} {\mathbf{z}}^{1} \ldots {\mathbf{z}}^{C} } \right]$. For an image having lesser than 6 salient regions, the position and texture features are set to zero for the missing ones
	e.	A secret key K_2_ randomly generates a row vector ${\mathbf{X}}_{2}$. The second intermediate hash $\left( {\mathbf{B}} \right)$ is formulated as ${\mathbf{B}} = \left[ {\left( {{\mathbf{B^{\prime}}} + {\mathbf{X}}_{2} } \right) {\text{mod }}256} \right]$
4.	Finally, the two intermediate hash vectors are concatenated and pseudo-randomly scrambled on the basis of secret key K_3_ to obtain the final hash $\left( {\mathbf{M}} \right)$	
	*Output: Final Hash*	

The length of the final hash has been discussed in Table 3. It is observed that the final hash length is 106 integers. As the dynamic range of integer is [0, 255], therefore, it requires 8 bit to represent each integer value. Hence, the final length of the hash in bits is 106 × 8 = 848 bits.

**Table 3:** Structure of image hash

Global vector	Salient vector S		
Ring partition-PGNMF	${\text{F}}\left( {x, y, w, h} \right)$	${\text{Z}}$	Final hash length
64 integers	4 × 6 = 24 integers	3 × 6 = 18 integers	106 integers

### Authentication of images

For an image authentication, we have the reference hash $\left( {{\mathbf{M}}_{0} } \right)$ of the original (reference) image, calculated hash (${\mathbf{M}}_{1} )$ for the received (test) image. The above two hashes are compared to check whether the received image is similar/tampered version of the original image or simply the different one. A pair of visually similar images may have different pixel values due to the content-preserving operations. But visually same are called similar or same image. The process of image authentication is as follows:

1. *Extracting the features* The final hash for the test image is calculated using the proposed algorithm as discussed in Table 2 and let the hash be named as ${\mathbf{M}}_{1} = \left[ {{\mathbf{Q}}_{1} \,\varvec{ }{\mathbf{F}}_{1} \,\varvec{ }{\mathbf{Z}}_{1} } \right].$
2. *Separation of final hash* The reference hash $\left( {{\mathbf{M}}_{0} } \right)$ obtained from the original image is decomposed into the global and local feature vectors to obtain the components ${\mathbf{Q}}_{0} , {\mathbf{F}}_{{0,\varvec{ }}}$ and ${\mathbf{Z}}_{0}$
3. *Saliency map comparison* The salient regions of the test image are compared with those of the original image using position vectors. If it is observed that the compared areas for a pair of salient regions are large enough, then it may be considered to be matched. Accordingly, the texture vectors are reshuffled by moving the matched components in each of the texture vector pairs ${\mathbf{Z}}_{0}$, ${\text{Z}}_{1}$.
  Consider an example where, there are five salient regions in the reference image and four in the test image.
  $${\mathbf{Z}}_{0} = \left[ {{\text{z}}_{0 }^{1} {\mathbf{z}}_{0 }^{2} {\text{z}}_{0}^{3} {\text{z}}_{0 }^{4} {\text{z}}_{0}^{5} 0 } \right]$$
  $$\varvec{ }{\mathbf{Z}}_{1} = \left[ {{\text{z}}_{1}^{1} \varvec{ }{\mathbf{z}}_{1}^{2} {\mathbf{z}}_{1}^{3} {\text{z}}_{1}^{4} 0 0 } \right]$$
  The first four pair of sub-vectors in ${\mathbf{Z}}_{0}$ and ${\mathbf{Z}}_{1}$ may be either match or unmatched. Accordingly, ${\mathbf{F}}_{0}$ and ${\mathbf{F}}_{1}$ are reshuffled.
4. *Calculating Euclidean distance and decision making* Euclidean distance between the image hashes is used to judge resemblance of the image pairs. Let us consider an image hash ${\mathbf{N}}$, which is structured by concatenating the global vector ${\mathbf{Q}}$ and the rearranged texture feature ${\mathbf{Z}}$, say ${\mathbf{N}} = \left[ {{\mathbf{Q}}, {\mathbf{Z}}} \right]$. Position feature $\left( {\mathbf{F}} \right)$ is not required for distance calculation. However, it will be used during the counterfeit area localization. The Euclidean distance between reference hash vector $({\mathbf{N}}_{0}$) and the test hash vector $({\mathbf{N}}_{1}$) is defined as:
  24 $${\text{D}} = \|{\mathbf{N}}_{1} - {\mathbf{N}}_{0}\|$$

The Euclidean distance is used to decide the similarities or dissimilarities with respect to the pre-determined threshold. In practice, the global feature of an image obtained by applying Ring partition-PGNMF approach is adequate enough to distinguish the similarities/dissimilarities of the image pairs. The inaccuracy of the saliency detection may have an undesirable influence on the calculation of Euclidian distance that may lead to erroneous results. To overcome this problem, the hash distance may be calculated based on global features only to detect the similarities/dissimilarities in the decision-making process. This is given as follows.

25 $${\text{D}} = \|{\mathbf{Q}}_{1} - {\mathbf{Q}}_{0}\|$$

## Results

The experimental results for the robustness, discriminative capability, and counterfeit area localization have been discussed in the next subsections.

### Robustness

Robustness evaluations are quantitative analysis for the normal content-preserving operations. An image hash should be approximately same after any non-malicious activity. These activities consist of image rotation, JPEG compression, watermark embedding, Gaussian low-pass filtering, gamma correction, brightness adjustment, contrast adjustment, and image scaling (Swaminathan et al. 2006).

We have tested the proposed model using 200 different images taken from various sources (USC-SIPI Image Database 2007; Ground Truth Database 2008). Because of space limitation, we have used only five images (Peppers, Airplane, House, Lena, and Baboon) for visual representation in the paper. Content preserving operation is created by StirMark 4.0 (Petitcolas 2000), Photoshop CS3 (10.0), and MATLAB (2013a) as shown in Table 4.

**Table 4:** Content-preserving operations with given parameter values

Tool	Manipulation	Parameter	Parameter values
Stir Mark	JPEG compression	Quality factor	30, 40, 50, 60, 70, 80, 90, 100
Stir Mark	Watermark embedding	Strength	10, 20, 30, 40, 50, 60, 70, 80, 90, 100
Stir Mark	Scaling	Ratio	0.5, 0.75, 0.9, 1.1, 1.5, 2.0
Stir Mark	Rotation and Cropping	Rotation angle in degree	1, 2, 5, 10, 15, 30, 45, 90, −1, −2, −5, −10, −15, −30, −45, −90
Photoshop	Brightness adjustment	Photoshop Scale	10, 20, −10, −20
Photoshop	Contrast Adjustment	Photoshop Scale	10, 20, −10, −20
Matlab	Gamma Correction	Y	0.5, 0.75, 0.9, 1.1, 1.5
Matlab	3 × 3 Gaussian low pass filter	Standard deviation	0.3, 0.4, 0.5, 0.6, 0.7, 0.8, 0.9, 1

**Fig. 7:**
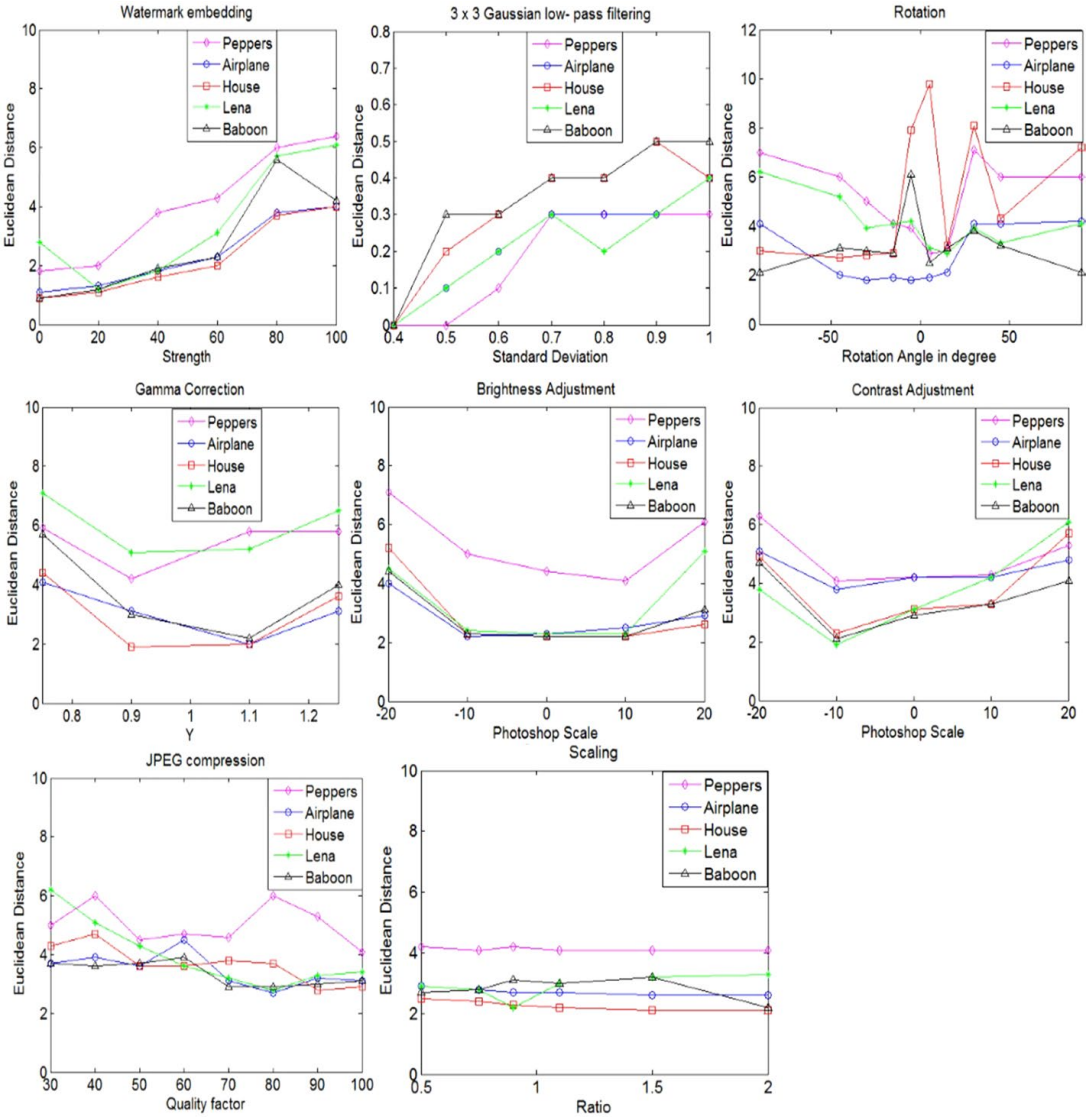
Robustness performance of the proposed algorithm for some content preserving operations

The Euclidean distance between the original image and the image after content preserving operations is shown in Fig. 7. It may be observed that if we choose the threshold of Euclidean distance as 7, then few perceptually similar image (i.e. 3/200 × 100% = 1.5%) may be considered as the different image, as shown in Fig. 7 (rotation operation). Therefore, the true rejection is 1.5%. However, the false acceptance is zero percent.

**Fig. 8:**
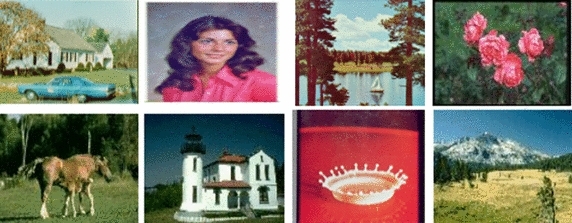
Different image samples used in discrimination test

**Fig. 9:**
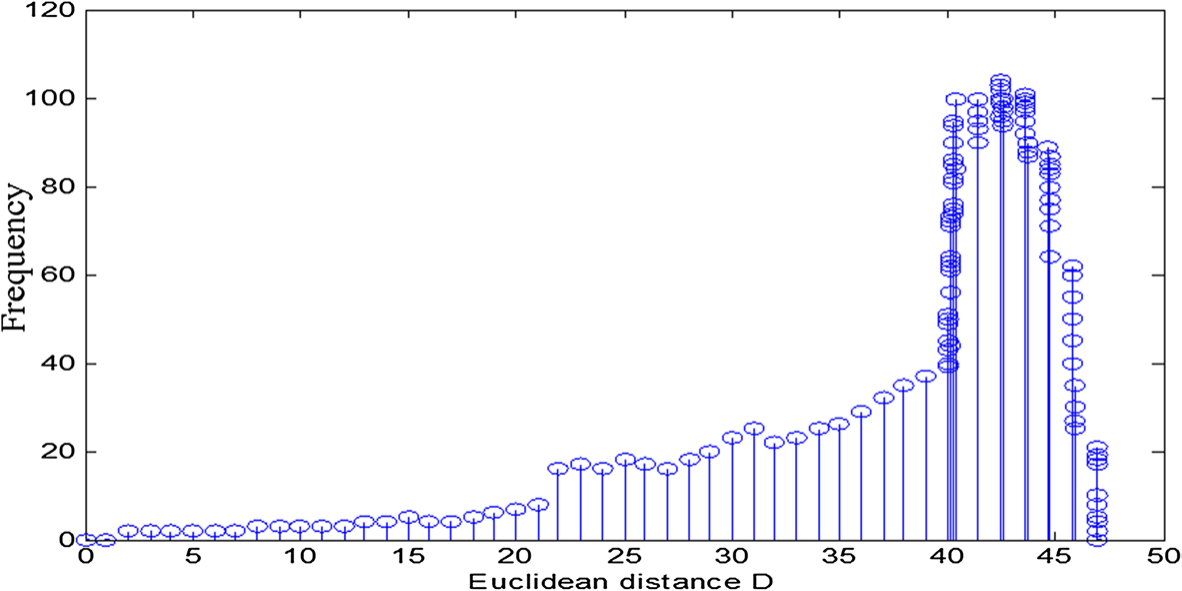
Discrimination test based on 300 different images

The Euclidean hash distances for visually different images are almost greater than 10 as shown in Fig. 9. If we increase the selected threshold of the Euclidean distance to 10, the true rejection is zero percent. Nevertheless, there are few cases (0.116%) in which the Euclidean distance is below 10 for different images. Thus, the false acceptance is 0.116%. Hence, it may be concluded that the threshold of the Euclidean distance may be selected according to the application of an image hashing.

For this study, we have used an image hashing technique for an image authentication purpose. The basic requirement of an image authentication is to have zero false acceptance rate. Because of this, we have selected a threshold Euclidian distance 7 for subsequent analysis. The experimental results which are shown in Fig. 7 demonstrates the effectiveness of the proposed method. It can be observed from Fig. 7, the Euclidean distances of some content preserving operations are less than the selected threshold (i.e. 7) except few cases in rotation operation. However, these few cases in rotation operation may be considered similar image at selected threshold 10, but with the cost of small false acceptance. Hence, the proposed method is robust to some content preserving operations.

### Discrimination capability

To check the discriminative capability of the proposed method for visually different images, we have taken 300 images from different sources. Some of the sample images (animal, car, house, scene and lady, etc.) from the 300 image set have been shown in Fig. 8. The hash vector for the 300 images has been generated using the proposed method and the calculated Euclidean distance for different possible combinations of hash vector pairs are shown in Fig. 9. It can be observed that the obtained Euclidean distances are above 7 (i.e. pre-determined threshold) in all the cases. This signifies that the proposed method provides satisfactory discrimination.

### Testing for counterfeit area localization

**Fig. 10:**
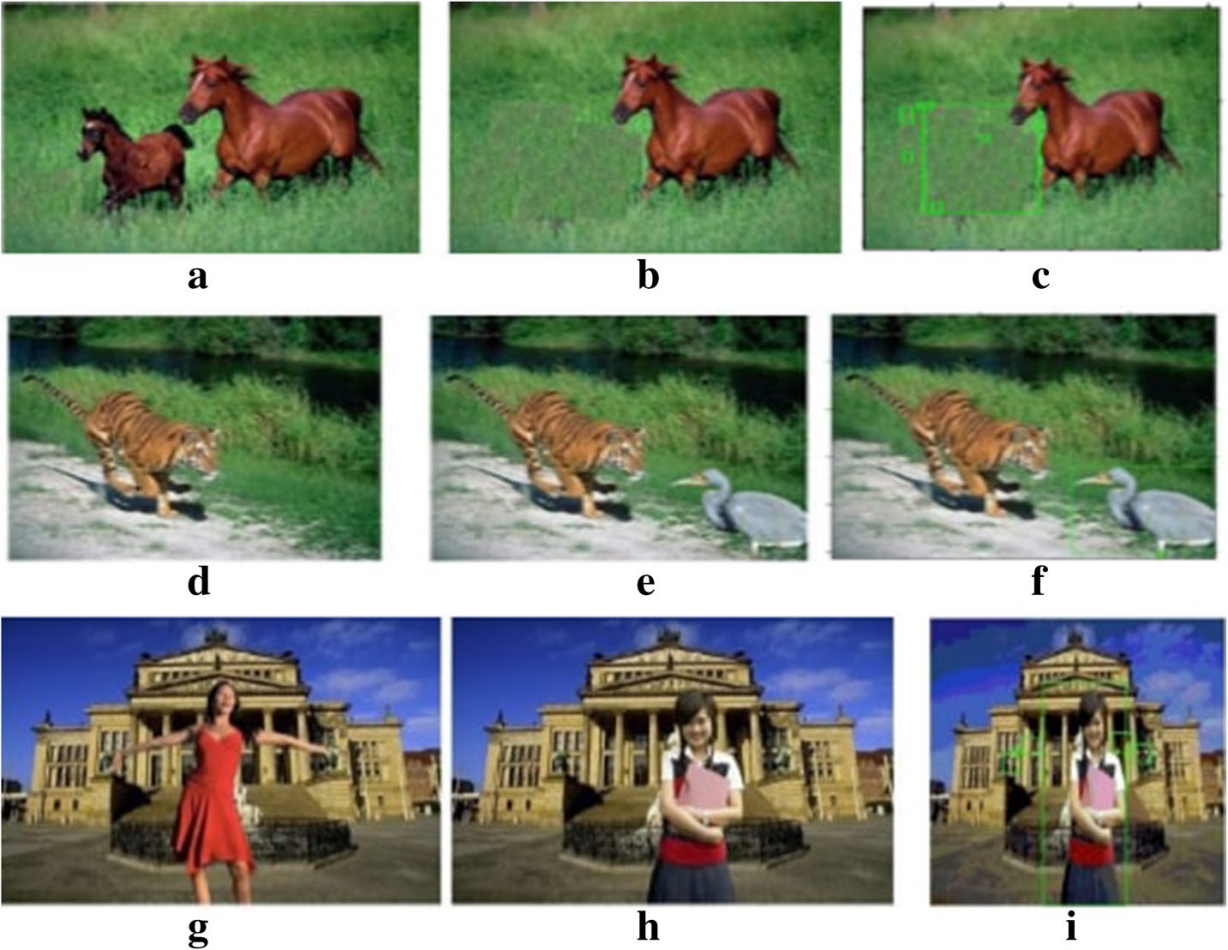
Forged area localization. **a**, **d**, **g** Original images. **b**, **e**, **h** Forged images. **c**, **f**, **i** *Green rectangle* signifies the localized forged area

In addition, we have carried out the experiments for malicious activities and localization of the counterfeit area. Whenever a particular image is attacked by the malicious operation, such as an insertion, deletion, and replacement, etc., it looks perceptually different (Swaminathan et al. 2006). Then, the obtained image is called a forged image and should provide different hash values. The forged images are generated using Photoshop. Some examples of the original and forged images are shown in Fig. 10. The experimental results of forged area localization have been shown in the third column of Fig. 10. In Fig. 10c, the green rectangle is the forged area, in which some content that was there in the original image has been removed in forged image. Similarly, green rectangle in Fig. 10f, i shows the forged area localization for content insertion and object replacement respectively. We have generated the hash vectors of original images and forged images, and calculated the hash distances which are shown in Table 5. It is observed that all the hash distance are more than the pre-selected threshold. Hence, it may be concluded that the proposed method is sensitive towards malicious changes made in the image.

**Table 5:** Image hashes for tampered image

Image pairs	D
Figure 10a, b	31
Figure 10d, e	29
Figure 10g, h	34

## Discussion

**Fig. 11:**
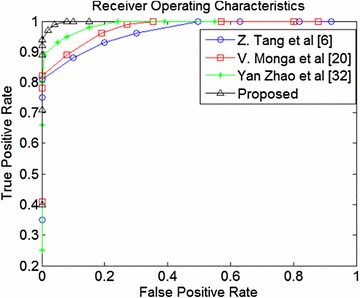
ROC curve comparisons of the different algorithms

The proposed algorithm has been compared with some of the well-known methods available in the literature (Ahmed et al. 2010; Khelifi and Jiang 2010; Monga and Mihcak 2007; Tang et al. 2008; Zhao et al. 2013). The performances have been evaluated using Receiver Operating Characteristics (ROC). The ROC is a characteristic curve plotted using false positive rate (FPR) along the abscissa and true positive rate (TPR) along the ordinate. The FPR and TPR are calculated as follows:

26 $${\text{FPR }} = \frac{{ \in_{1} }}{{O_{1} }}$$

27 $${\text{TPR }} = \frac{{ \in_{2} }}{{O_{2} }}$$

where, $\in_{1}$ represents the number of visually different image pairs judged as similar image pairs and $\in_{2}$ represents the number of perceptually identical image pairs judged as similar image pairs. The *O*_1_ and *O*_2_ denote the total number of visually dissimilar image pairs and a total number of perceptually similar image pairs respectively. The ROC comparison amongst the different algorithms is shown in Fig. 11. It can be observed from Fig. 11 that the proposed method TPR is higher especially at low FPR as compared to the other methods.

The performance comparison of the proposed method with some of the state-of-the-art methods are shown in Table 6. The methods proposed in Ahmed et al. (2010), Monga and Mihcak (2007), Tang et al. (2008) are not robust to the rotation operation. This may be due to the block-based operations carried out for feature extraction. The hashing methods proposed in Tang et al. (2008) and Monga and Mihcak (2007) fails to localize the forged area in the image. The method proposed in Khelifi and Jiang (2010) is robust to content-preserving operations but fails to find the small counterfeit area. The method proposed in Zhao et al. (2013) is robust against almost all the geometric operations but not robust to rotation operation beyond 5°. For ROC comparison, we have used only the baseline methods (used in this study) as described in Monga and Mihcak (2007), Tang et al. (2008), Zhao et al. (2013).

**Table 6:** Comparison of proposed method with some of the state-of-the-art methods

Comparison parameters	Ahmed et al. (2010)	Tang et al. (2008)	Monga and Mihcak (2007)	Khelifi and Jiang (2010)	Zhao et al. (2013)	Proposed method
Features used	Local	Global	Local	Local	Global and local features	Global and local features
Hash Length	7168 bits	320 bits	64 floating point numbers	250 bits	560 bits	848 bits
Robust against JPEG compression	Y	Y	Y	Y	Y	Y
Robust against rotation	NA	NA	NA	Y	Up to 5° only	All Arbitrary degree
Ability to detect small area forgery	Y	Y	Y	NA	Y	Y
Capability to find the counterfeit regions	Y	NA	NA	NA	Y	Y
Optimal TPR When FPR = 0	0.7843	0.8101	0.8213	0.8943	0.9157	0.9811
Optimal FPR When TPR = 1	0.3265	0.4972	0.3545	0.6427	0.2187	0.0012
Average time (s)	NA	0.9321	2.98	2.6	2.4	2.1

*NA* not applicable, *Y* Yes

It may be observed from Table 6 that the proposed algorithm is robust against the image manipulation and possesses good discriminating characteristics. Moreover, it can locate the counterfeit area with sufficient accuracy. Table 6 shows that the proposed method TPR (i.e. 0.9811 with zero FPR) and FPR (i.e. 0.0012 with TPR one) is higher and lower respectively as compared to other methods. The limitation of the proposed algorithm is that full image information has not been used for hash generation. Around 21% of the image information remain unused during the conversion from a square image into a secondary image. The other drawback is that the forged area cannot be accurately located under simultaneous rotation and malicious operation being carried out in the image.

The computational complexity of the proposed method and some of the state-of-the-art methods have been evaluated on a personal computer (with Intel core i7, 3.40 GHz, 8 GB RAM) running MATLAB 2013a. The comparison is shown in Table 6. It is observed that the proposed method is computationally efficient as compared to some of the existing methods. The reason for increased computational complexity as compared to the system proposed in Tang et al. (2008) may be due to the use of local features along with global features. Even though the computation complexity is not mentioned in Ahmed et al. (2010), it may be inferred that the major computation load is not because of wavelet transform but due to determining the decision threshold using various image processing operations.

## Conclusions

In this paper, we have proposed a robust image hashing based on global features extracted through ring partition-PGNMF and local features that consist of position and texture information. The combination of global and local features is robust against the content-preserving operations, which has a desirable discriminative capability also. The local feature is useful to localize the forged area. The enhancement of the discriminating capability of proposed hashing is due to PGNMF because it is good at learning part based representations. The PGNMF converges faster as compared to other NMFs. Experiments have been carried out on large image dataset to evaluate the performance of proposed hashing method. It is observed that proposed image hashing technique is robust to content preserving operations. It has also the capability to localize the forged area with sufficient accuracy.

The proposed algorithm may not be able to detect the color changes in the image. In future, the efficient color feature may be considered to detect the color forgery. Forged area localization under simultaneous rotation and malicious operations may also be explored. The other limitations such as reduction of hash length and computational complexity may be addressed during the future scope of work.

## Abbreviations

DWT: discrete wavelet transform
DFT: discrete Fourier transform
MD: message digest
SHA: secure hash algorithm
DCT: discrete cosine transform
NMF: non-negative matrix factorization
